# Supplementary material for: Increased Epidermal Nerve Growth Factor without Small-Fiber Neuropathy in Dermatomyositis
Source: Int J Mol Sci. 2022 Aug 12;23(16):9030. doi: 10.3390/ijms23169030 (PMC9408946; doi:10.3390/ijms23169030)
Supplement: Supplementary file 1 [file ijms-23-09030-s001.zip › ijms-1842232-supplementary.pdf]

Table S1. Demographic data from patients with dermatomyositis.

Patient No.	Age (year)	Gender	Biopsied location
DM01	68	F	face
DM02*	52	M	face
DM03	55	F	face
DM04	66	M	face
DM05	67	M	face
DM06	48	F	chest
DM07	85	M	chest
DM08	45	M	neck
DM09	67	F	back
DM10	63	F	back
DM11	85	M	back
DM12	85	M	abdomen
DM13	58	F	neck
DM14	43	M	neck
DM15	52	M	back
DM16	37	F	upper limb
DM17	63	M	upper limb
DM18	58	F	finger
DM19	69	F	upper limb
DM20	56	F	upper limb
DM21	65	M	upper limb
DM22	40	F	lower limb
DM23	71	F	lower limb
DM24	86	M	lower limb
DM25	57	M	upper limb
DM26	54	F	upper limb
DM27	68	F	dorsal hand
DM28	57	F	upper limb
DM29	70	M	upper limb
DM30	25	F	lower limb
DM31	55	F	upper limb
DM32	80	M	hand
DM33	77	F	upper limb
DM34	86	M	upper limb
DM35	55	M	finger

* Sample were used for the representative immunofluorescent images in the figures.

F, female; M, male.

Table S2. Demographic data from patients with cutaneous lupus erythematosus.

Patient No.	Age (year)	Gender	Biopsied location	Type of LE	SLE
LE01	58	F	face	DLE	-
LE02	58	F	face	DLE	+
LE03	55	F	face	DLE	+
LE04	38	F	face	SCLE	-
LE05	56	F	face	Lupus profundus	+
LE06	40	M	face	DLE	-
LE07*	24	M	face	DLE	-
LE08	51	F	face	ACLE	-
LE09	38	F	face	DLE	+
LE10	16	M	face	ACLE	+
LE11	38	F	face	ACLE	+
LE12	56	M	face	DLE	-
LE13	69	M	face	SCLE	-
LE14	34	M	face	DLE	+
LE15	67	M	face	DLE	-
LE16	66	F	face	ACLE	+
LE17	68	F	face	DLE	-
LE18	51	F	face	ACLE	+
LE19	47	F	face	DLE	-
LE20	51	M	chest	SCLE	-
LE21	50	F	chest	DLE	-
LE22	56	M	back	DLE	-
LE23	31	F	abdomen	SCLE	-
LE24	22	F	palm	ACLE	+
LE25	55	F	upper limb	ACLE	+
LE26	45	F	upper limb	SCLE	-
LE27	31	F	lower limb	DLE	-
LE28	28	F	hand	ACLE	+
LE29	54	F	upper limb	DLE	-

* Sample were used for the representative immunofluorescent images in the figures.

F, female; M, male; ACLE, acute cutaneous lupus erythematosus; DLE, discoid lupus

erythematosus; SCLE, subacute cutaneous lupus erythematosus; SLE, systemic lupus erythematosus.
